# Identifying Frequent Health Care Users and Care Consumption Patterns: Process Mining of Emergency Medical Services Data

**DOI:** 10.2196/27499

**Published:** 2021-10-06

**Authors:** Laura Maruster, Durk-Jouke van der Zee, Erik Buskens

**Affiliations:** 1 Faculty of Economics and Business University of Groningen Groningen Netherlands; 2 Health Technology Assessment, Department of Epidemiology University Medical Center Groningen Groningen Netherlands

**Keywords:** process mining, frequent users, hospital care, emergency medical services, regional care networks, elderly, Netherlands

## Abstract

**Background:**

Tracing frequent users of health care services is highly relevant to policymakers and clinicians, enabling them to avoid wasting scarce resources. Data collection on frequent users from all possible health care providers may be cumbersome due to patient privacy, competition, incompatible information systems, and the efforts involved.

**Objective:**

This study explored the use of a single key source, emergency medical services (EMS) records, to trace and reveal frequent users’ health care consumption patterns.

**Methods:**

A retrospective study was performed analyzing EMS calls from the province of Drenthe in the Netherlands between 2012 and 2017. Process mining was applied to identify the structure of patient routings (ie, their consecutive visits to hospitals, nursing homes, and EMS). Routings are used to identify and quantify frequent users, recognizing frail elderly users as a focal group. The structure of these routes was analyzed at the patient and group levels, aiming to gain insight into regional coordination issues and workload distributions among health care providers.

**Results:**

Frail elderly users aged 70 years or more represented over 50% of frequent users, making 4 or more calls per year. Over the period of observation, their annual number and the number of calls increased from 395 to 628 and 2607 to 3615, respectively. Structural analysis based on process mining revealed two categories of frail elderly users: low-complexity patients who need dialysis, radiation therapy, or hyperbaric medicine, involving a few health care providers, and high-complexity patients for whom routings appear chaotic.

**Conclusions:**

This efficient approach exploits the role of EMS as the unique regional “ferryman,” while the combined use of EMS data and process mining allows for the effective and efficient tracing of frequent users’ utilization of health care services. The approach informs regional policymakers and clinicians by quantifying and detailing frequent user consumption patterns to support subsequent policy adaptations.

## Introduction

A large part of regional health care consumption is attributed to “frequent users” (ie, patients who make repeated calls to hospital and nursing health care services) [1]. Although definitions differ, a threshold of 4 to 5 calls or more per year is generally used to classify a patient as a “frequent user” [2,3]. While frequent users represent a minority of emergency department (ED) patients (4.5%-8%), they may account for up to 21%-28% of all ED visits [4]. Frequent users appear heterogeneous as a group; however, they may be clustered into distinct categories relating to their health care needs and the health services provided to them [5].

The high workload and costs incurred by frequent users make them a relevant target group for regional policymakers and clinicians to consider as they attempt to make the best use of scarce resources. For example, due to the various health care needs related to advanced age, the “frail elderly” are known to be frequent users [6-9]. Their frailty is related to their status of being extremely vulnerable to endogenous and exogenous stressors, exposing them to a higher risk of negative health-related outcomes [8]. Importantly, they are often confronted with fragmented health care [6], inappropriate or delayed triage at EDs [10], and incorrect referrals. These observations suggest an inappropriate approach to their health care needs and the potential unnecessary use of health care services. Once their frailness is identified, advanced health care planning may be used to improve their health care continuity [8,11,12]. Therefore, tracing frequent users and their (shared) consumption patterns is a prerequisite for assessing the efficiency and effectiveness of current clinical practice, undertaking appropriate actions to improve it, and evaluating the added value of these actions and related policy changes.

Notably, many frequent users do not make calls to a single health care provider but are network users, making consecutive visits to multiple health care providers. Apart from their preferences, their network use may be explained, for example, by specialized health care needs. Thus, tracing frequent users requires a network-based approach, including all regional health care providers. Unfortunately, collecting data by interviewing health care providers or even extracting data from their local records tends to be cumbersome. Rules on patient privacy, competition among health care providers, incompatibility of information systems, and the effort required may present hurdles that are not easily overcome, adding to data collection costs. Most research designs limit their scope to single or related health care providers, with a primary focus on hospitals [13]. As a result, many frequent users may be overlooked. Moreover, their routings along different health care providers may appear fragmented due to a lack of information on major health care providers outside hospitals, such as nursing homes, which are particularly relevant to the frail elderly.

This article explores an alternative approach to tracing frequent users, relying on emergency medical services (EMS) data (ie, records of ambulance rides containing patient and logistic data). Acting as the “ferrymen” in the regional health care network, EMS theoretically direct subacute or acute patient routings, starting with a time-ordered sequence of ambulance rides. Therefore, their databases potentially offer an efficient means for identifying and tracing frequent users. Importantly, the EMS patient population is likely to accommodate many frequent users [14,15]. Moreover, patients served by EMS tend to require substantial health care resources, as indicated by their need for mobile nursing services and transport. However, the potential of using EMS data to identify frequent users and their consumption patterns has hardly been acknowledged in the literature [16].

This study aimed to show how the analysis of EMS records may contribute to tracing frequent users on a regional scale, especially the frail elderly, and reveal their health care consumption patterns. The novel analysis technique of process mining is instrumental to the study, enabling the automated identification of patient routings (ie, identifying health care providers consulted over time by combining the records related to ambulance rides). Frequent users can be identified by quantifying their number of ambulance rides via process mining. In turn, their consumption patterns are reflected in their routings, specifying health care providers and specialties involved over time. The aggregation of patient routings establishes trends in their annual demand for health care and the associated workload distribution over the network. Process mining has an advantage over other mapping techniques as it uses factual observations retrieved from data rather than man-made process models. Process mining has been successfully used to analyze health care processes, usually in an intrahospital context [17-27]. However, its application to regional health care networks is new.

As relevant background information, we briefly summarize that the Dutch system for chronic healthcare has for a long time been funded through national funds under the General Act Special Care Costs [28]. However, this funding scheme was deemed too inefficient and generous. Accordingly, as of January 1, 2015, a major system change was introduced, accompanied by new legislation (ie, the Long-term Care Act ) [29]. The execution and implementation of health care for less severe indications in the home setting were transferred to the municipalities to achieve a better match and a more efficient system. The transfer was expected to result in more elderly people remaining in their homes, thus reducing chronic (elderly) health care costs. As of spring 2021, these expectations, to a large extent, have not materialized or even worsened the situation. Our study reveals problems that have emerged since the system’s change, including subsequent nursing home closures.

Using EMS records of the province of Drenthe, the Netherlands, this study shows how ambulance data allows for effective and efficient tracing and quantification of frequent users of health care services on a regional scale, considering frail elderly users as a focal group. The proposed approach builds on the role of EMS as the regional “ferryman,” implying the utilization of a single key source for data collection, covering many health care providers at the same time (ie, hospitals, nursing homes, and EMS). Process mining adds to the efficiency of the approach by enabling automated mapping of patient routings (ie, their consecutive visits to health care providers). Furthermore, the structure of patient routes is analyzed at patient and group levels, allowing us to gain insight into regional coordination issues and workload distributions among health care providers, which is helpful to policymakers and clinicians. In particular, we seek evidence for the effects of the Dutch governmental policies described above, including the impact of higher health care need entry requirements for nursing home admissions on regional health care consumption [30-32].

## Methods

### The Health Care Network of the Province of Drenthe

The province of Drenthe has a population of 491,867, with a population density of 183 inhabitants per square kilometer [33]. Hospital care is provided by 4 hospitals within the province and by several hospitals located in neighboring provinces. Among the 4 hospitals, 3 offer basic treatment, and 1 hospital has the necessary skills and resources to treat multilevel trauma. The reasons for referral to hospitals outside the province include being close to the patient scene, patient preferences, level of health care, or specialization related to specific treatments. Nursing care is also provided by a few dozen nursing homes, mainly located within the province. EMS is provided by a single operator, relying on a network of 17 bases in 14 towns or villages in Drenthe. Its services include both urgent (A-rides) and planned (B-rides) patient transport to hospitals and planned (B-rides) transport to nursing homes. B-rides are legitimized by patient health care needs prohibiting self-transport or transport by taxi. A-rides assume the presence of staff and equipment, enabling advanced life support (ALS; ie, advanced health care for critical patients). In contrast, B-rides may be offered with either ALS or basic life support, setting less strict medical skillsets and equipment requirements.

### Data

Patient data were collected from EMS records of ambulance rides performed between January 1, 2012, and December 31, 2017. The data collected included the ride dates and times, destinations (ie, health care providers), patient age, urgency (A or B), and health care providers’ medical specialty as indicated by the patient’s health care needs. A unique identification number assigned to each patient identified patients’ routings along different health care providers and services by combining their records and organizing them according to ambulance ride dates.

Based on our focus on the frail elderly population, frequent users, and EMS scope of services, 3 categories of health care providers were distinguished: hospitals, nursing homes, and the EMS. The latter was considered a formal health care provider when the treatment provided by the ambulance paramedics on the scene sufficed to address patient health care needs, designated EMS “see and treat” (EMS-S&T). This inclusion may also serve as an indicator of inappropriate or fragmented health care [6].

### Process Mining

Like many regions worldwide, the province of Drenthe has increasing numbers of frail elderly people who utilize a significant part of the regional health care system. In terms of their visits to regional hospitals and nursing homes, their consumption patterns remain largely unknown, as regional health care providers do not disclose this type of patient information.

Process mining is a technique combining data science and process management to support the identification and analysis of operational processes (ie, sequential activities undertaken by an organization in satisfying its customers), thereby relying on event logs (ie, recordings of respective activities) stored in a database [34]. Process mining has been successfully used to map health care processes, clarifying how patients are served as a net effect of activities performed by health care providers. So far, most examples of its use in health care are related to an intrahospital context [17-27]. By automatically generating process maps using factual observations retrieved from data, process mining has important advantages over other modeling techniques that rely on manual observations of the actual system or inspection of documents [17,34].

EMS records referring to single rides are anonymized, cleaned by removing empty records (ie, records not relating to patients), and inspected for data accuracy to allow for process mining. If a record lacks information on the health care provider (eg, the destination of a ride), it was marked “unknown.” Subsequently, Disco (version 2.2.0; Fluxicon) [35], a tool used to perform the process mining, traced patient routings by combining and ordering (time-wise) patient records referring to unique patients.

The health care consumption of frequent users (ie, patients meeting a threshold of 4 ambulance rides to regional health care providers within a year) is quantified by presenting their numbers and the number of calls, including annual trend figures and the distribution of frequencies. Frail elderly users (patients 70 years of age or more) were considered a subgroup among frequent users. They were analyzed for urgency using the ambulance ride categories A (urgent) and B (planned) as a proxy and for the volume of recognized categories of frequent users among them, specifically patients known to be in frequent need of dialysis, radiation therapy, or hyperbaric medicine (DRH). The “known” patient categories depend on local insight, thereby relying on EMS staff and records. For “unknown” patient categories, such a classification is lacking.

The health care consumption of frail elderly users was assessed at two levels (the patient level and the patient group level) using process mining for structural analysis. At the patient level, patient routings along the various health care providers are identified by the ride destinations, including the specialties being consulted. Patients are distinguished by two categories (low complexity and high complexity) as indicated by their routings. This complexity is considered from the perspective of health care consumption uncertainty. For instance, low-complexity patients are the “known” patients, involving few health care providers, and high-complexity patients are those for whom routings might not be fully understood. Although the term “low complexity” might suggest that these patients have a relatively mild health condition, they are frail and consume many resources and should also be recognized as a relevant subgroup.

### Ethics Approval and Consent to Participate

Since the data originally were routinely collected for administrative purposes and completely anonymized, this study does not fall within the scope of the Medical Research Involving Human Subjects Act (Wet Medisch-weteschappelijk Onderzoek 2021) [31]. Accordingly, we obtained a full waiver for using anonymized data from the EMS services from the Medical Ethics Review Board of the University Medical Center Groningen (reference number METc 2018/402).

## Results

### Quantifying Frequent Users

#### Overview

Table 1 provides an overview of all patients served by regional health care providers based on EMS records. In total, 126,758 unique patients were identified between 2012 and 2017, involving 212,967 calls for services and omitting 2494 records not linked to patients. Table 1 shows call volumes have increased 25% over the years, from 31,300 to 39,235 calls. Similar changes are observed for frequent users' general health care consumption, including frail elderly users. Frequent users account for approximately 16% of total regional health care consumption, of which more than half is attributable to the frail elderly. The number of frail elderly users increased from 395 to 628 (59%), and their calls rose from 2607 to 3615 (39%). Strikingly, the largest growth in frail elderly users was observed from 2013 to 2015, increasing from 320 to 548 (71%) patients.

**Table 1:** Overview of patients served by regional health care providers.

Year	All patients, N	All patient calls, N	Frequent users, n	Frequent users, n (%)^a^	Frail elderly, n	Frail elderly, n (%)^b^
2012	22,551	31,300	731	5051 (16)	395	2607 (52)
2013	23,794	32,359	625	4636 (14)	320	2428 (52)
2014	24,355	34,568	844	5681 (16)	446	2792 (49)
2015	25,677	36,742	987	5976 (16)	548	3169 (53)
2016	27,146	38,763	999	6449 (17)	561	3333 (52)
2017	27,671	39,235	1043	6258 (16)	628	3615 (58)
Total	151,194 (126,758)^c^	212,967	5229 (4734)^c^	34,051	2898 (2700)^c^	17,944

^a^Calls made by frequent users as a percentage of calls from all patients.

^b^Calls made by frail elderly users as a percentage of calls from frequent users.

^c^Number of unique patients involved.

#### Frail Elderly

Details on the health care needs for frail elderly users and their urgency are shown in Table 2, which are categorized by distinguishing their EMS calls according to the medical specialty requested and the urgency of the ride (Tables 1 and 2). For example, among the 395 frail elderly users in 2012 (Table 1), 302 (76%) patients had been transported at least once in an urgent ride (A), while 345 (87%) patients had used at least 1 planned ride (B; Table 2). In addition, among patients transported in planned rides, 75 (19%) patients required dialysis, radiation therapy, or hyperbaric medicine (B-DRH), and 326 (83%) patients had other diverse health care needs and urgencies (B-other). The 2 patient groups overlap due to comorbidity; therefore, the sum of their patient numbers exceeds the overall annual number of frail elderly users. The right-hand side of Table 2 shows the number of calls, indicating the number of rides associated with the groupings mentioned above.

Table 2 reveals the number of urgent (A) calls among frail elderly users more than doubled between 2012 and 2017 (from 825 to 1729, 110%), in contrast to the modest growth in calls for rides planned in advance (B; from 1782 to 1886, 6%). In addition, between 2012 and 2017, the number of frail elderly users requiring either specific, predictable treatment or other treatments (Table 2, see columns B-DRH and B-other) increased from 75 to 104 (39%) and 326 to 487 (49%), respectively. However, annual calls made by DRH patients diminished between 2012 and 2017 (from 728 to 502, –31%), while a considerable growth in the number of calls made had occurred for the remaining group (from 1054 to 1384, 31%).

**Table 2:** Health care needs and urgency for frail elderly users.

Year	Frail elderly population								Frail elderly calls						
	All, N	Urgency of health care, n			Health care needs, n			All, N			Urgency of health care, n			Health care needs, n	
	A^a^+B^b^	A	B	B-DRH^c^		B-other^d^	A+B			A		B	B-DRH		B-other
2012	395	302	345	75		326	2607			825		1782	728		1054
2013	320	258	271	64		256	2428			664		1764	903		861
2014	446	372	370	74		353	2792			1019		1773	740		1033
2015	548	473	408	83		395	3169			1478		1691	533		1158
2016	561	498	417	99		398	3333			1552		1781	575		1206
2017	628	561	501	104		487	3615			1729		1886	502		1384
Total	2898 (2700)^e^	2464 (2303)^e^	2312 (842)^e^	499 (480)^e^		2,215 (2119)^e^	17,944			7267		10,677	3981		6696

^a^A: urgent transport.

^b^B: planned transport.

^c^B-DRH: patients in need of dialysis, radiation therapy, or hyperbaric medicine making use of EMS planned transport.

^d^B-other: patients with health care needs other than dialysis, radiation therapy, or hyperbaric medicine making use of EMS planned transport.

^e^Number of unique patients involved.

### Identifying Regional Health Care Consumption Patterns Among Frail Elderly Users

#### Patient Level: Low- and High-Complexity Patients

**Figure 1:**
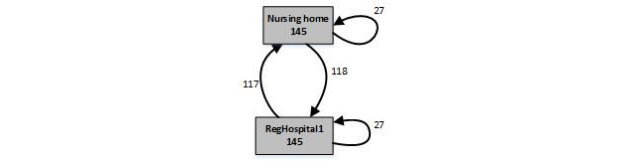
The process map of low complexity patient A.

**Figure 2:**
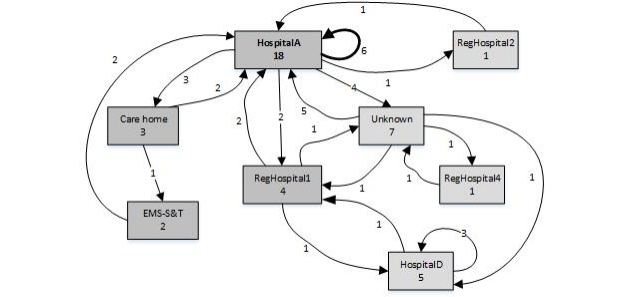
The process map of high complexity patient B. EMS-S&T: emergency medical services “see and treat.”

Consumption patterns for frail elderly users are captured by ordered ride lists and process maps (Tables 3 and 4; Figures 1 and 2). Tables 3 and 4 show an excerpt of the routings of 2 frequent users, patient A and patient B, respectively. Patient A needs dialysis and is served by a single hospital (RegHospital 1). Patient A’s routing exhibits low complexity, which is clearly shown by the process map in Figure 1. It illustrates how the patient was treated 145 times by RegHospital 1 while living in the nursing home between 2012 and 2017. Arcs in Figure 1 summarize information on the sequence of services consumed; for example, a ride to RegHospital 1 is directly followed by a ride to the nursing home 117 times, and a ride to the nursing home is directly followed by a ride to RegHospital 1 118 times. In some cases, transport to or from the hospital has not been organized by the EMS provider under study. For instance, a ride to RegHospital 1 is followed by another ride to RegHospital 1 27 times. Similarly, a ride to the nursing home is followed by another ride to the nursing home 27 times.

**Table 3:** Excerpt from the routings of patient A.

Date	Time	Urgency	Destination	Specialty
2012-01-06	16:06:15	B	Nursing home	Other specialties
2012-01-19	15:46:06	B	Nursing home	Internal medicine
2012-01-20	09:30:56	B	RegHospital 1-dialysis	Other specialties
2012-01-23	10:15:32	B	RegHospital 1-dialysis	Other specialties
2012-01-23	15:16:49	B	Nursing home	Internal medicine
2012-01-25	09:11:57	B	RegHospital 1-dialysis	Other specialties

**Table 4:** Excerpt from the routings of patient B.

ID	Date	Time	Urgency	Activity	Specialty
1	2013-09-13	06:50:39	A1	EMS-S&T^a^	OS^b^
2	2013-06-02	02:42:14	A1	Hospital A	OS
3	2013-06-16	13:50:06	A2	Hospital A	OS
4	2013-07-02	00:04:43	A1	Hospital A	P^c^
5	2014-02-01	20:40:56	A2	Hospital A	OS
6	2014-02-01	23:09:41	A2	Unknown	OS
7	2014-11-26	22:26:42	A1	Hospital A	OS
8	2014-12-06	11:22:00	A1	Hospital A	P
9	2014-12-08	12:51:39	A1	Hospital A	Surgery
10	2014-12-08	16:14:38	B	Unknown	OS
11	2014-02-17	11:42:49	A1	Hospital A	P
12	2014-04-02	09:37:52	B	RegHospital 2	OS
13	2015-09-05	08:43:23	A1	RegHospital 4	General
14	2015-09-05	11:04:53	B	Unknown	Unknown
15	2015-09-23	01:51:15	A2	Hospital D	General
16	2015-10-22	07:57:29	A2	Hospital D	General
17	2015-02-06	09:56:39	A2	Hospital A	P
18	2015-02-06	11:56:25	A2	Nursing home	P
19	2015-02-12	11:05:34	A1	Hospital A	P
20	2015-02-12	12:47:12	B	Nursing home	P
21	2015-02-26	08:57:59	B	Hospital A	OS
22	2015-02-26	10:47:32	B	Nursing home	OS
23	2015-01-07	08:16:58	A1	Hospital A	OS
24	2015-01-07	10:02:35	A1	Unknown	OS
25	2015-01-07	11:48:10	A1	RegHospital 1	Surgery
26	2015-03-15	05:22:40	A1	EMS-S&T	OS
27	2015-01-11	16:09:12	A2	Hospital A	OS
28	2015-01-11	17:46:08	B	RegHospital 1	OS
29	2015-04-30	14:39:22	A2	Hospital A	P
30	2015-04-30	16:02:48	B	Unknown	P
31	2015-01-14	08:55:44	B	Hospital A	P
32	2015-06-23	16:47:33	A2	Hospital A	P
33	2015-06-23	19:00:12	B	Unknown	P
34	2015-06-28	10:43:34	A1	Hospital A	General
35	2015-06-28	21:01:32	A1	RegHospital 1	P
36	2015-07-10	11:45:13	A1	Hospital D	General
37	2015-07-12	12:09:24	A1	RegHospital 1	IM^d^
38	2015-07-12	14:21:53	B	Unknown	IM
39	2015-01-21	21:39:06	A2	Hospital A	P
40	2016-01-14	08:58:03	A2	Hospital D	P
41	2016-01-02	22:08:36	A1	Hospital D	P

^a^EMS-S&T: emergency medical services “see and treat.”

^b^OS: other specialties.

^c^P: pulmonology.

^d^IM: internal medicine.

Whereas routings for patient A exhibit low complexity, other patients may have more complex routings, as illustrated by patient B in Table 4 and Figure 2. Patient B is among the very frequent users (between 2012 and 2017), with 41 ambulance rides and diverse health care needs as indicated by the specialties attending to the patient’s treatment, thus relying on several health care providers.

#### Patient Group Level: “Unknown” Patients

**Figure 3:**
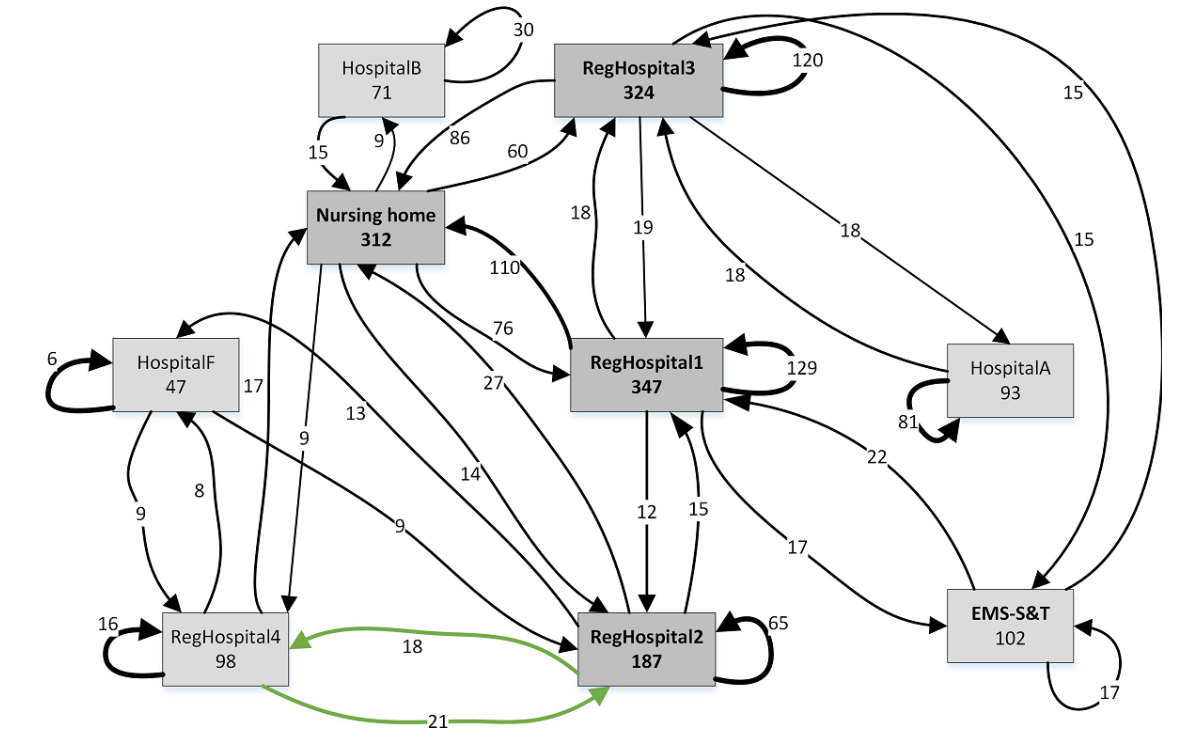
Process maps of 2012 aggregated routings for frail elderly patients (non-dialysis, radiation therapy, or hyperbaric medicine). EMS-S&T: emergency medical services “see and treat.”

**Figure 4:**
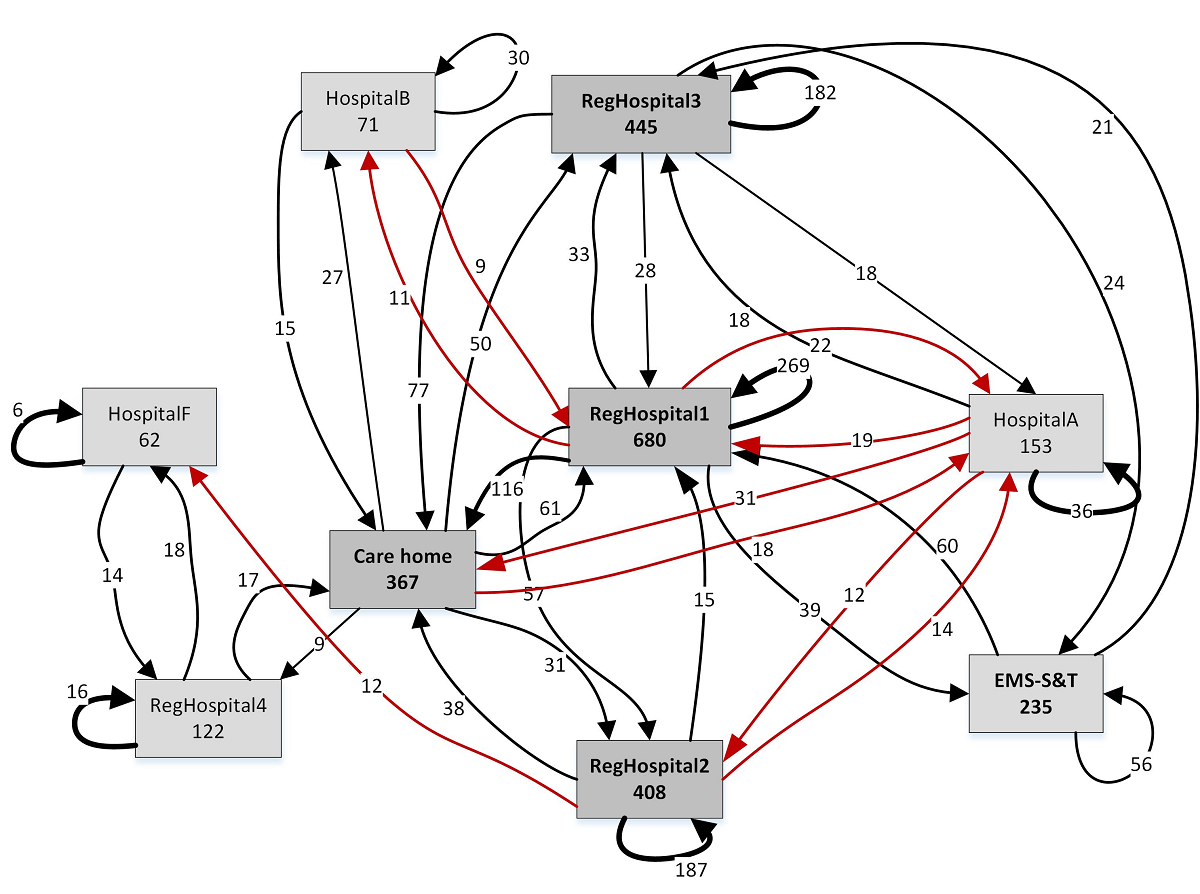
Process maps of 2017 aggregated routings for frail elderly patients (non-dialysis, radiation therapy, or hyperbaric medicine). EMS-S&T: emergency medical services “see and treat.”

Figure 3 and Figure 4 show the workload distribution for frail elderly users who did not belong to a known category for 2012 (326 patients) and 2017 (487 patients; Table 2, see column B-other). Only the health care providers involved in at least 30 treatments and had arcs with frequencies of at least 8 are shown. The number of treatments provided by all health care providers rose considerably between 2012 (Figure 3) and 2017 (Figure 4), although the growth rate is quite different across health care providers. This is paralleled by the higher connectivity among health care providers in 2017, as indicated by the arc frequencies and new arcs (Figure 4, see arcs marked in red). However, developments were not necessarily unidirectional, as connections may disappear over the years (Figure 3, see arcs marked green).

## Discussion

### Principal Findings

Frequent users, defined as patients making repeated calls to regional health care services, have a high impact on health care capacities and resource management. Tracing frequent users and their (shared) consumption patterns may be instrumental in regional policymaking. In this study, we combined EMS records and process mining in the Dutch province of Drenthe to trace frequent users and discern different types of users. The approach allowed us to develop and assess patient routings along various regional health care providers by combining their records on ambulance rides. This study demonstrates that this approach can effectively and efficiently trace and quantify frequent users and assess their consumption patterns. Considering frail elderly users as a focal group, the effectiveness of the approach benefits from its broad scope, spanning a large group of health care providers, including nursing homes. Moreover, using EMS records as a single source of data guaranteed the availability of data on all regional hospitals. Notably, data collection and analysis costs were low as the approach relied on a single data source that is routinely collected and the use of automated data analysis by process mining.

Process mining of EMS records confirms the relevance and impact of frail elderly users as a subgroup of frequent users [6-8], representing over 50% of frequent users and meeting a threshold of 4 calls in 1 year. Moreover, the number of frail elderly users and the number of calls they made to health care providers significantly increased during the observation period. Strikingly, the largest growth in frail elderly users was observed in 2014 and 2015, increasing by 100 patients a year, from 320 in 2013 to 548 patients in 2015 (71%). Also, the urgency of their calls increased as indicated by a steep increase in the number of urgent rides in this category (from 664 in 2013 to 1478 in 2015, 123%), substantially exceeding the background annual growth rate of EMS transports of 5.1% over the observation period [30,36].

Our observations parallel structural changes in the Dutch healthcare system of elderly health care (Wet Maatschappelijke Ondersteuning 2015) [37]. Closure of health care homes and stricter health care need entry requirements for nursing homes forced the elderly to continue living independently in their homes for as long as possible [32,38,39]. Although the respective transformations intended to reduce chronic health care costs, they resulted in more hospital admissions and acute situations that were no longer manageable in the home setting, which indeed may be considered counterproductive. We feel the structural change in the trend observed in EMS figures for 2014 and 2015 may indicate an unwanted and unexpected impact of national policy changes. Quantifying the health care consumption of the frail elderly population may be helpful to policymakers by showing their impact on the system and revealing the urgency to address their needs. Furthermore, understanding the health care consumption of the frail elderly can lead to discussions about residential and other health care homes or other forms of home health care. Our findings reveal a need for further action, such as capacity extensions, tailored home health care services, or advanced health care planning to improve elderly health care and its coordination.

Based on process mining, the structural analysis of patient consumption patterns revealed 2 patient groups: low-complexity “known” patients who require DRH and “unknown” patients often linked to complex routings and the use of several regional health care providers. Importantly, while the known group of frail elderly patients exhibited a growth rate mirroring demographic changes, the unknown group exhibited growth at a much higher pace, implying increased and unpredictable workloads. The workload related to the latter group tends to be increasingly distributed over the several regional health care providers, requiring them to become better connected over time to provide the best health care for the patients they jointly serve. This may be explained by ongoing regional specialization, calling for regional coordination in identifying and addressing patient needs and managing capacities. As a result, process mining further identifies the health care providers involved and the nature of their involvement in terms of specialties.

Of patients with high call frequencies, we evaluated 2 specific cases and noted that some of these patients might be considered low complex. For example, patient A needed frequent dialysis treatments but was mainly served by a single health care provider. Alternatively, patient B was associated with multiple health care providers. The latter patient may benefit from scrutinizing their treatment plan and organizing advanced health care planning if deemed necessary. However, using this method to identify such cases would assume that potential privacy issues are recognized and resolved, which is beyond the scope of this paper.

Policymakers and clinicians may use the results of our analyses to engage in discussions or assess the current standard of care. Our results indicate that frequent users with no clear indication, such as dialysis, are “shopping and hopping,” representing an unmet need while utilizing excessive resources. The onus is on general practitioners and nursing home specialists to address this challenge. Advanced care planning and timely and appropriate care at the right location for this category of frequent users might enhance their quality of life while saving scarce resources. Providing sound evidence for the latter would require a different type of study.

### Limitations

This study also has limitations. Firstly, only the frail elderly were studied at some depth, using EMS records for only one province in the Netherlands. Nevertheless, while health care consumption patterns are likely to be affected by regional characteristics, it is expected that the success of the proposed approach is not dependent on the latter. Secondly, EMS records only include frequent users who are not capable of self-transport. Thus, frequent users who do not or rarely use EMS will not be traced by the proposed approach. Thirdly, as it is explorative, the paper highlights the potential of the proposed approach in tracing frequent users and enhancing regional policymaking. Ongoing and future research should be directed toward confirming and expanding the method, including comparisons with alternative approaches.

### Conclusions

The combined use of EMS data and process mining allows for the effective and efficient tracing of frequent users of health care services. The approach supports regional policymakers and clinicians by quantifying and detailing frequent user consumption patterns to support subsequent policy adaptations.

## Abbreviations

ALS: advanced life support
DRH: dialysis, radiation therapy, or hyperbaric medicine
ED: emergency department
EMS: emergency medical services

## References

[1] Hudon C, Courteau J, Krieg C, Vanasse A (2017). Factors associated with chronic frequent emergency department utilization in a population with diabetes living in metropolitan areas: a population-based retrospective cohort study. BMC Health Serv Res.

[2] Urbanoski K, Cheng J, Rehm J, Kurdyak P (2018). Frequent use of emergency departments for mental and substance use disorders. Emerg Med J.

[3] Seguin J, Osmanlliu E, Zhang X, Clavel V, Eisman H, Rodrigues R, Oskoui M (2018). Frequent users of the pediatric emergency department. CJEM.

[4] LaCalle E, Rabin E (2010). Frequent users of emergency departments: the myths, the data, and the policy implications. Ann Emerg Med.

[5] Cook LJ, Knight S, Junkins EP, Mann NC, Dean JM, Olson LM (2004). Repeat patients to the emergency department in a statewide database. Acad Emerg Med.

[6] Dollard J, Harvey G, Dent E, Trotta L, Williams N, Beilby J, Hoon E, Kitson A, Seiboth C, Karnon J (2018). Older People Who Are Frequent Users of Acute Care: A Symptom of Fragmented Care? A Case Series Report on Patients' Pathways of Care. J Frailty Aging.

[7] Komenda P, Tangri N, Klajncar E, Eng A, Di Nella M, Hiebert B, Strome T, Lobato de Faria R, Zacharias JM, Verrelli M, Sood MM, Rigatto C (2018). Patterns of emergency department utilization by patients on chronic dialysis: A population-based study. PLoS ONE.

[8] Legramante JM, Morciano L, Lucaroni F, Gilardi F, Caredda E, Pesaresi A, Coscia M, Orlando S, Brandi A, Giovagnoli G, Di Lecce VN, Visconti G, Palombi L (2016). Frequent Use of Emergency Departments by the Elderly Population When Continuing Care Is Not Well Established. PLoS ONE.

[9] Burns TR (2017). Contributing factors of frequent use of the emergency department: A synthesis. International Emergency Nursing.

[10] Rutschmann OT, Chevalley T, Zumwald C, Luthy C, Vermeulen B, Sarasin FP (2005). Pitfalls in the emergency department triage of frail elderly patients without specific complaints. Swiss Med Wkly.

[11] Goldstein J, McVey J, Ackroyd-Stolarz S (2015). The Role of Emergency Medical Services in Geriatrics: Bridging the Gap between Primary and Acute Care. CJEM.

[12] Kue R, Ramstrom E, Weisberg S, Restuccia M (2009). Evaluation of an emergency medical services-based social services referral program for elderly patients. Prehosp Emerg Care.

[13] Chan JS, Tin AS, Chow WL, Tiah L, Tiru M, Lee CE (2017). Frequent attenders at the emergency department: an analysis of characteristics and utilisation trends. Proceedings of Singapore Healthcare.

[14] Edwards MJ, Bassett G, Sinden L, Fothergill RT (2015). Frequent callers to the ambulance service: patient profiling and impact of case management on patient utilisation of the ambulance service. Emerg Med J.

[15] Scott J, Strickland AP, Warner K, Dawson P (2014). Describing and predicting frequent callers to an ambulance service: analysis of 1 year call data. Emerg Med J.

[16] Brown JF, Raven MC, Tangherlini NL, Kennedy Hall M (2018). Frequent Users of 9-1-1 Emergency Medical Services: Sign of Success or Symptom of Impending Failure?. Prehosp Emerg Care.

[17] Mans RS, van der Aalst WMP, Vanwersch RJB (2015). Process Mining in Healthcare: Evaluating and Exploiting Operational Healthcare Processes.

[18] Rebuge Á, Ferreira DR (2012). Business process analysis in healthcare environments: A methodology based on process mining. Information Systems.

[19] Rovani M, Maggi FM, de Leoni M, van der Aalst WM (2015). Declarative process mining in healthcare. Expert Systems with Applications.

[20] Delias P, Doumpos M, Grigoroudis E, Manolitzas P, Matsatsinis N (2015). Supporting healthcare management decisions via robust clustering of event logs. Knowledge-Based Systems.

[21] Pika A, Wynn MT, Budiono S, Ter Hofstede AHM, van der Aalst WMP, Reijers HA (2020). Privacy-Preserving Process Mining in Healthcare. Int J Environ Res Public Health.

[22] Pereira GB, Santos EAP, Maceno MMC (2020). Process mining project methodology in healthcare: a case study in a tertiary hospital. Netw Model Anal Health Inform Bioinforma.

[23] Lu F, Li P, Bao Y, Liu C, Zeng Q (2020). Death Risk Prediction of Intensive Care Unit Patients Combined with Treatment Process Mining. J Med Imaging Hlth Inform.

[24] Duma D, Aringhieri R (2018). An ad hoc process mining approach to discover patient paths of an Emergency Department. Flex Serv Manuf J.

[25] Orellana A, Davila C, Leon I (2019). Detection of Bottlenecks in Hospital Processes from the XAVIA HIS System using Process Mining. IEEE Latin Am. Trans.

[26] Ibanez-Sanchez G, Fernandez-Llatas C, Martinez-Millana A, Celda A, Mandingorra J, Aparici-Tortajada L, Valero-Ramon Z, Munoz-Gama J, Sepúlveda M, Rojas E, Gálvez V, Capurro D, Traver V (2019). Toward Value-Based Healthcare through Interactive Process Mining in Emergency Rooms: The Stroke Case. IJERPH.

[27] Kurniati A, Hall G, Hogg D, Johnson O (2018). Process mining to explore variation in chemotherapy pathways for breast cancer patients. British Journal of Cancer.

[28] Overheid.nl (2014). [translated from Dutch: General Act Special Medical Expenses (A.W.B.Z.)]. Algemene Wet Bijzondere Ziektekosten.

[29] Overheid.nl (2021). Wet langdurige zorg.

[30] Nederlandse_Zorgautoriteit (2018). Monitor acute zorg.

[31] Overheid.nl (2021). Wet medisch-weteschappelijk onderzoek.

[32] Koopmans RT, Pellegrom M, van der Geer ER (2017). The Dutch Move Beyond the Concept of Nursing Home Physician Specialists. J Am Med Dir Assoc.

[33] Eurostat (2019). Eurostat - Population projections.

[34] van der Aalst W (2012). Process Mining. ACM Trans. Manage. Inf. Syst.

[35] Fluxicon (2019). Disco.

[36] Rijksoverheid.nl (2019). Kamerbrief over stand van zaken Ambulancezorg en rol in acute zorg.

[37] Overheid.nl (2015). Wet maatschappelijke ondersteuning 2015.

[38] Kroneman M, Boerma W, van den Berg M, Groenewegen P, de Jong J, van Ginneken E (2016). Netherlands: Health System Review. Health Syst Transit.

[39] Maarse JAMH, Jeurissen PPP (2016). The policy and politics of the 2015 long-term care reform in the Netherlands. Health Policy.

